# Combination of stem cell therapy and acupuncture to treat ischemic stroke: a prospective review

**DOI:** 10.1186/s13287-022-02761-y

**Published:** 2022-03-03

**Authors:** Huidong Jia, Jia He, Lan Zhao, Chia-Chen Hsu, Xiaofeng Zhao, Yuzheng Du, Lin Han, Zhanfeng Cui, Xuemin Shi, Hua Ye

**Affiliations:** 1 The Oxford Suzhou Centre for Advanced Research (OSCAR), Building A, 388 Ruo Shui Road, Suzhou Industrial Park, Jiangsu, People’s Republic of China; 2 First Teaching Hospital of Tianjin University of Traditional Chinese Medicine, 88 Chang Ling Road, Xi Qing District, Tianjin, People’s Republic of China; 3 National Clinical Research Center for Chinese Medicine Acupuncture and Moxibustion, 88 Chang Ling Road, Xi Qing District, Tianjin, People’s Republic of China; 4 Department of Engineering Science, Institute of Biomedical Engineering, University of Oxford, Roosevelt Drive, Oxford, OX3 7DQ UK

**Keywords:** Ischemic stroke, Stem cell therapy (cytotherapy), Acupuncture, Combination therapy

## Abstract

Stroke is the second leading cause globally that leads to severe disability and death. Stem cell therapy has been developed over the recent years to treat stroke and diminish the mortality and disability rate of brain injuries. Acupuncture, which can activate endogenous recovery via physical stimuli, has been applied to enhance the recovery and rehabilitation of stroke patients. Attempts have been made to combine stem cell therapy and acupuncture to treat stroke patients and have shown the promising results. This prospective review will look into the possible mechanisms of stem cell therapy and acupuncture and intend to undercover the potential benefit of the combined therapy. It intends to bridge the modern emerging stem cell therapy and traditional acupuncture at cellular and molecular levels and to demonstrate the potential benefit to improve clinical outcomes.

**Supplementary Information:**

The online version contains supplementary material available at 10.1186/s13287-022-02761-y.

## Background

Stroke is the second leading cause of mortality and disability of adults all over the world, which caused 5.5 million deaths globally in 2016 [1]. In adults, ischemic stroke represents about 87% of stroke cases [2]. The pathophysiologic processes of brain neural tissue death and vasogenic edema occurring in ischemic stroke result from exposing the brain to reduced oxygen levels (hypoxia) and/or blood supply (ischemia), and the secondary excitotoxicity and oxidative stress in the acute phase, following the apoptosis and inflammatory damage in the subacute phase, resulting in dyskinesia, aphasia, sensory disturbances, ataxia sequelae symptoms, or even death [3, 4].

**Fig. 1:**
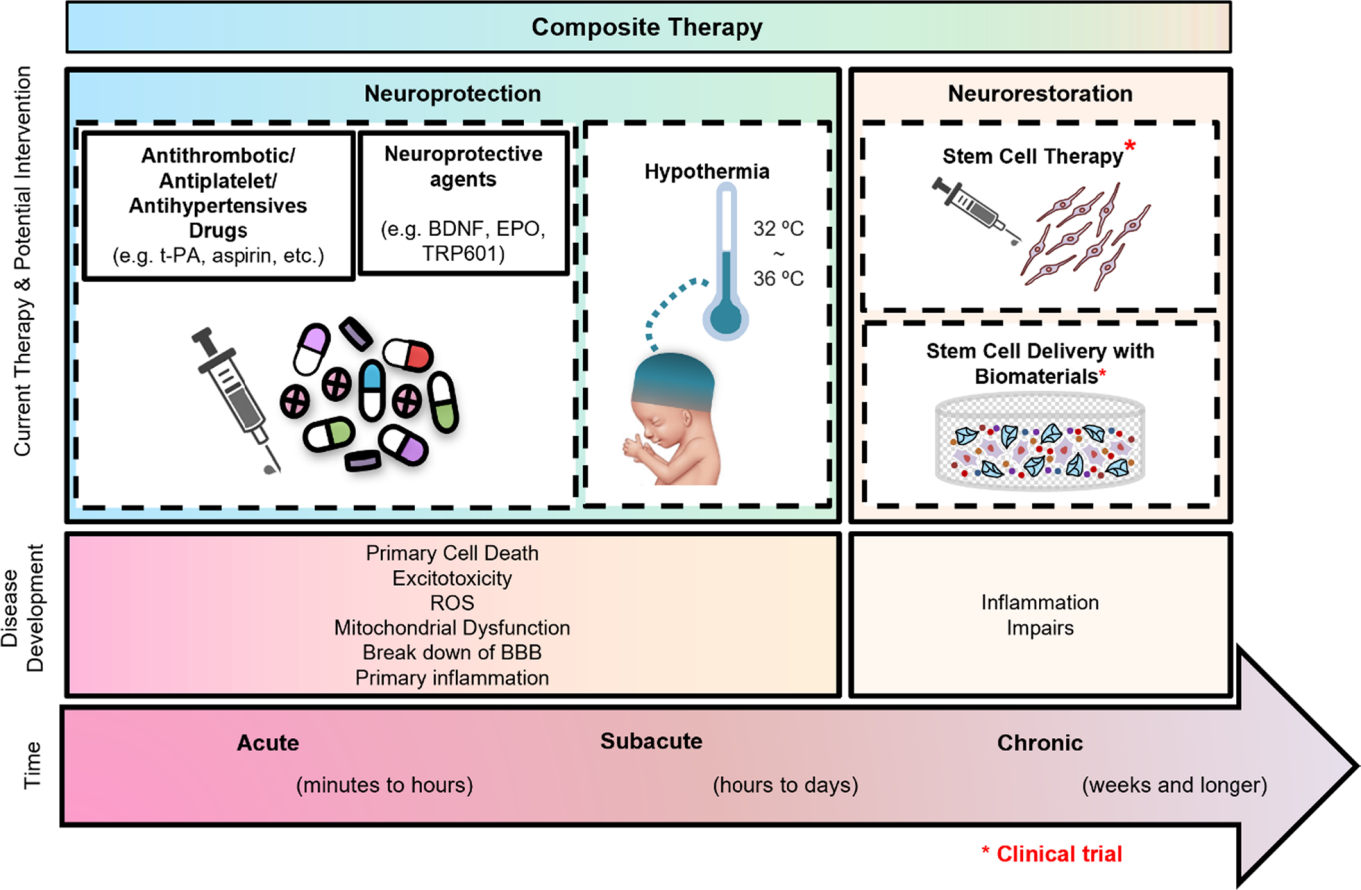
Current therapy for ischemic stroke. Antithrombotic medication will be given to an acute phase patient within hours post-stroke, together with neuroprotective agents and hypothermia treatment to diminish neural cell death and to prevent further inflammatory damage. Stem cell therapy is applied in the chronic phase for the purpose of neuro-restorations in multiple clinical trials. *BBB* blood–brain barrier, *BDNF* brain-derived neurotrophic factor, *EPO* erythropoietin, *ROS* reactive oxygen species

Current treatments for stroke mainly focus on neuroprotection and recanalizing obstructed cerebral blood vessels, which include hypothermia and the administration of antithrombotic, antiplatelet, and antihypertensive drugs for ischemic brain damage caused by an embolism (Fig. 1) [5–7]. Although these treatments could partly prevent brain injury progression through reduction of metabolic demands, suppression of excitotoxicity, and free radical activity [8], the 6-h rescue time window limits the efficiency, and none of the treatments can promote neuro-regeneration, which aims to replace the dead neurons and to rebuild a functional neuronal network in order to contribute to behavioral improvements [9, 10].

In the last two decades, stem cells, with their capability to self-renew and differentiate into multiple cell derivatives, have shed light on treating stroke [5]. A wide variety of different stem cell types have been used in experimental and clinical studies in a variety of applications, including cell replacement, activation of endogenous neurogenesis and angiogenesis, neuroprotection, and modulation of inflammation and immune responses for the regeneration of the lost cells and enhancement of neurogenesis to improve long-term recovery [11–13].

Acupuncture has been used in stroke management for more than thousands of years in China. Random controlled clinical trials showed that acupuncture ameliorate post-stroke paralysis, ataxia, shoulder pain, aphasia, and dysphagia, promoting rehabilitation and reducing fatality rates [14–19].

An overview of stem cell therapy and acupuncture for stroke treatment and the mechanisms behind their effects will be summarized in this review, which aims to explore their intrinsically cooperative therapeutic effects. Clinical trials using cell-based therapies and acupuncture for stroke patients will be evaluated and a series of cellular products currently under development will be highlighted. Finally, we will discuss the benefit and possibility of developing a new stroke therapeutic strategy combing implantation of functional cells and acupuncture as a potential complementary therapy to other conventional treatments. The administration protocol for clinical stem cell therapy, for example the cell dose or administration route of the therapy, is not the focus of this study due to the lack of sufficient clinical data for randomized controlled trials (RCTs).

## Stem cell therapy for ischemic stroke

Studies of stem cell therapy for ischemic stroke have mostly focused on two different mechanisms: (1) replacement of damaged neural cells and tissues and (2) paracrine functional effects including immunomodulation, pro-angiogenesis, and neuroprotective and neurotrophic functions [20, 21]. Broadly, embryonic stem cells (totipotent stem cells), fetal stem cells (mainly the fetal brain or spinal cord derived-neural stem cells), adult stem cells (tissue-specific stem cells, such as mesenchymal stem cells), and induced pluripotent stem cells (genetically engineered stem cells) are the most preclinically and clinically tested cell types in the ischemic stroke regenerative therapy. The clinical trials of cell therapy for stoke are summarized in the Additional file 1.

### Cell types in stroke cytotherapy (Table 1)

Embryonic stem cells (ESCs) are derived from the inner cell mass of the blastocyst and are characterized by self-renewal and the ability to differentiate into cell derivatives of all three germ layers. In particular, clinically relevant neural lineage cells, such as cortical neurons, motor neurons, astrocytes, oligodendrocytes, and other neural cell types, have successfully been derived from human ESCs [21–23]. However, ethical concerns and tumorigenesis are the major obstructs hindering the ESCs from entering human clinical trials.

**Table 1:** Clinical trials of stem cell-based therapies to treat ischemic stroke

Cell type	Advantage	Disadvantage	Administration Route	Implantation windows	References
MSCs	Abundant, Low ethical concern, Low immunogenicity, Multi-paracrine effects	No neural cell or tissue regeneration ability	All available route (IV, IA, IT, IP)	Acute phase, Less than 6 months	[50–52]
NSCs	Neural cell replacement, Neural tissue regeneration	Limited sources and expansion ability, Ethical concern	Intracerebral	Chronic phase, Not earlier than 1 month	[35, 53]
iPSCs/iNSCs	Sufficient neural cells, No ethical concern, Paracrine effects	Genetically modified, Tumorigenesis risk, Immunogenicity	NRG—Intracerebral PE—any route	NRG—chronic phase, PE—acute phase	[36, 54]

*NRG* neural regeneration, *PE* paracrine effect

IV, intravenous; IA, intraarterial; IT, intrathecal; IP, intraperitoneal; MSCs, mesenchymal stem cells; NSCs, neural stem cells; iPSCs, induced pluripotent stem cells; iNSCs, immortalized NSCs

Induced pluripotent stem cells (iPSCs) are reprogramed from somatic cells, such as fibroblasts, into a stem cell state with self-renewal capacity and pluripotency similar to ESCs. However, unlike ESCs, iPSCs can bypass certain ethical issues and can be used to derive patient-specific cells, which can lower the risk of immune rejection [24]. There has been extensive research on robust protocols to generate neuronal and glial lineages from human iPSCs [25–27]. iPSCs has been recognized as one of the best regenerative medicine resources if the tumorigenesis risk could be removed or under control.

Neural stem cells (NSCs) sources can be endogenous or exogenous. Cells used to generate endogenous NSCs are adult NSCs located in the subventricular zone, the hippocampal dentate gyrus, and the olfactory bulb [11]. Previously, studies have shown that a limited number of endogenous NSCs can be activated and recruited to an infarcted area to promote neuro-regeneration [28]. Exogenous NSCs are derived from exogenous pluripotent stem cells (as discussed above in the ESCs and iPSCs sections), bone marrow-derived multipotent stem cells, or isolated from fetal and adult nervous systems [29–31]. Preclinical and clinical trials of commercialized engineered NSC lines, such as NSI-566 (human fetal spinal cord-derived NSC line) from *Neuralstem Inc* and CTX cell line (immortalized NSC line from brain frontal cortex tissue by c-mycER^TAM^ technique) from *ReNeuron* Ltd., have reported promising outcomes for treating stroke [32, 33].

Mesenchymal stem cells (MSCs) are multipotent stem cells that can be derived from a wide range of tissues, commonly including umbilical cord blood, umbilical cord, placenta, bone marrow, and adipose tissues. They are a mixture of multi-cell populations with origin tissue-specific properties but share a similar phenotype and a plastic adherent proliferation pattern [34]. Although MSCs have been shown to differentiate into neural lineages in some studies, the neural regeneration mechanism behind this differentiation has mainly been attributed to trophic effects of angiogenesis, neurogenesis, as well as modulating the host immune response [35].

Other cell types: Perinatal tissues have become one of the major stem or progenitor cell sources, which are abundant and without ethical concerns [36, 37]. Mononuclear cells (MNCs) isolated from umbilical cord blood (UCB), comprising a mixture of more than three subpopulations of hematological stem cells (HSCs), endothelial progenitor cells (EPCs), and MSCs, have also shown some positive effects for ischemic stroke therapy in animal models and human clinical trials [38–41]. EPCs can differentiate into mature endothelial cells, which exert pro-angiogenesis effects and have been observed migrating to the boundary zones of ischemic infarcted areas of Sprague–Dawley rat brains when intravenously administered within 24-h post-middle cerebral artery occlusion (MCAO). EPCs were found to incorporate into blood vessels in ischemic tissues [42], indicating their potential for use in stroke cell therapies [43].

### Therapeutic mechanism of stem cell therapy

In the early stage of cerebral ischemia, the main cause of the destruction of brain tissues is a cascade of damage due to anoxic depolarization, excitotoxicity, oxidative stress, and necrosis. Protecting cells in the peri-infarct area from excitotoxicity and oxidative stress is of the highest priorities for the treatment in the early-stage of ischemic brain injury [44].

#### Antioxidation and ionic homeostasis

MSCs possess a great potential for treating early-stage ischemic brain injury because of their paracrine functions, tolerance, and adaptive capacity to the brain ischemic and hypoxia microenvironment. Kaneko et al. demonstrated a successful combinatorial treatment of hypothermia and MSCs for neuron protection in vitro and proposed the delta opioid pathway is a therapeutic mechanism of the stem cell therapy, which maintains ionic homeostasis and endogenous neuroprotection [45].

The mechanisms of stem cell paracrine functions in antioxidative properties have been studied by analyzing the antioxidant defense and scavenging effects of stem cell-conditioned media. Modulating the signaling pathway AKT/pAKT and ERK1/2/pERK, activating the antioxidant proteins (such as Keap1, Nrf2, and HO-1), and releasing the neurotrophic factors NGF and BDNF have been reported from different in vitro studies [46, 47]. Lee et al. also discovered that oxidative stress and glial activation level decreased in rats with Alzheimer's disease after receiving hUCB-MSC implantation, which resulted in learning and memory function improvement [48].

Pathological membrane hyperpolarization prevents correct functioning of the electron transport chain, resulting in mitochondrial failure, which is one of the major steps leading to BBB dysfunction, focal vascular destruction, and progressive neural cell death in the acute stages of ischemic stroke. Intercellular mitochondrial transfer has been identified as an important mechanism of tissue regeneration by providing a means to improve metabolism in damaged cells [49]. Boukelmoune et al. found the NSCs that uptake the labeled MSCs' mitochondria have more intact mitochondrial membranes and better mitochondria function in a co-culture system that contains neurotoxic factors, which suggests that the transferring of mitochondria exerts additional cell recovery benefits beyond the direct uptake of intact organelles [50].

#### Immunomodulation

MSCs have been demonstrated to be able to systemically suppress over-responsive immune reactions by modulating the production of pro- or anti-inflammatory factors and the activity of immune cells. IFN-γ and IL-1 are crucial pro-inflammatory factors that can activate MSC immune‐suppressive effects [51, 52]. The immunomodulatory effects of MSCs, via regulating T cell, B cell, dendritic cell, and natural killer (NK) cell activity, have been shown to prevent deleterious autoimmunity in ischemic tissues [53, 54]. The MSCs implanted in the acute phase have been found to reduce the size of necrotic area and maintain motor functions through downregulating M1 macrophage/microglia activation and decreasing infiltration of *γδ* T cells, while increasing the presence of CD4 + *T*_regs_ and *T*_reg_-associated cytokines, which play a neuroprotective role via secretion of anti-inflammatory cytokines like IL-10 [55, 56].

Another major mechanism by which MSCs modulate the immune response is establishing negative-feedback loops to suppress inflammatory activity and promote tissue regeneration. MSCs can activate M2 macrophages to produce anti-inflammatory cytokines IL-10 and perform phagocytic activity by producing cyclooxygenase 2 and indoleamine 2,3-dioxygenase, therefore, reducing neutrophil immersion and diminishing further damage to the injured tissue [57].

#### Promoting vascular remodeling

BBB plays a pivotal role in maintaining the homeostasis of central nervous system (CNS) as a protective semipermeable shelter, regulating the exchange of substances between the circulating blood and the brain [58]. The loss of BBB integrity is associated with worse stroke outcomes, leading to vasogenic edema, brain swelling, and even cerebral hemorrhage [59].

Implantation of MSCs and/or EPCs could aid in the reconstruction of astrocytic end-feet and tight junctions, via the secretion of proangiogenic growth factors, including vascular endothelial growth factor (VEGF), basic fibroblast growth factor (FGF-2), and transforming growth factor-beta (TGF-β), attenuation of immune cell infiltration, MMP9 downregulation, and VEGF-A signaling pathway modulation effects, to restore the integrity of BBB and the functional recovery of the cerebral vasculature at the acute phase of ischemic stroke [60–62].

#### Pro-angiogenesis

A key factor for neural regeneration and the restoration of cerebral function in stroke tissues in the post-acute phase is revascularization of the necrotic zone and penumbra. Although both MSCs and EPCs produce necessary angiogenic factors, such as those in the FGF and VEGF families, MSCs are not directly involved in vasculature reconstruction like EPCs, but perform immunomodulation in the infarct region [63, 64]. Kang et al. revealed that pericytes coverage ratio is associated with angiogenesis with the use of a biomimetic vasculogenic model, which suggests that replenished multi-functional cells play a crucial role in balancing the immune response and revascularization in ischemic stroke therapy [65].

#### Impaired neural tissue replacement

Gliosis and gliotic scars enclose the necrotic infarct area at the post-stroke stage and the permanent neural tissue loss causes a series of neural dysfunctional symptoms. Only a limited number of endogenous NSCs can be activated by the signaling factors secreted from the lesion area to promote neuro-regeneration [11, 28]. To achieve better neuroprotection and neurogenesis, researchers have genetically modified NSCs (gm-NSC) or applied iPSC-derived NSCs in neuroregenerative studies. Zhang et al. reported that intravenously transplanted bFGF-expression NSCs can survive, migrate, and differentiate into neurons and glial cells in a MCAO rat model. Their histological analysis indicated that endogenous NSCs are activated and involved in neural tissue regeneration [66]. Along with the extensive preclinical studies on ischemic stroke, a commercial gm-NSC line (CTX, ReNeuron Ltd.) has been used in the first-in-man trial via stereotactic ipsilateral putamen injection to treat patients that are 6–60 months after ischemic stroke. This open-label, single-site, dose-escalation study showed no adverse effects with improved neurological function [32].

### Stem cell therapy strategy and limitations in ischemic stroke treatment

Stem cell therapy is considered a promising treatment for degenerative diseases and organ impairment. The clinical trials of cell therapy for stroke are summarized in Additional file 1: Supplementary Table 1. However, their curative effects on ischemic stroke are controversial as most of the relative clinical trials did not achieve the therapeutic targets that have been proved in animal studies. How to ensure the survival rate, maintain cell survival in vivo, improve target organ homing efficiency, differentiation ability, and paracrine functionality of the implanted stem cells are the major challenges in stem cell therapy [67, 68].

The means by which cells are delivered to stroke patients are selected based on the stage of the stroke and the desired therapeutic outcome. Multipotent stem cells are administered systemically via intravenous, intraarterial, intrathecal, and intraperitoneal routes when treatment via their paracrine effects is desired. Although systemic injection is a safe and convenient method to treat early-stage ischemic stroke patients, lung, liver, kidney, and spleen may clear or retain most of the implanted cells. Moreover, BBB prevents most of the leftover circulating stem cells from reaching the infarcted zone [13, 38].

Stem cell cerebral stereotactic implantation is an alternative cell treatment solution to improve neuro-regeneration, developed following the progression of live-imaging technology (which improves the accuracy) and biomaterial technology (which increases the cell survival and homing rate by providing better cell carriers, such as injectable hydrogels). However, direct injection of stem cells into the brain is a risky procedure and can only be performed during the chronic stage of a stroke, after the infarction scar has already formed and the intracranial pressure returns to normal [69, 70].

Despite of the revolutionary equipment and technologies developed, it has remained a challenge to deliver cells to the intracerebral microenvironment of ischemic stroke patients due to the complexity of pathophysiological causes. Other factors resulting in low intracerebral migration and low homing rate are not due to immune rejection, but are due to ischemia, inflammation, oxidative stress in the wounded area and the presence of endogenous electric fields within the patient body [71]. In particular, high levels of ROS and free radicals in and around the infarcted area of the brain can induce cell death and dysfunction of the administered stem cells [72].

## Acupuncture to treat ischemic stroke

Acupuncture has been served as an optional treatment in stroke rehabilitation. Clinical and laboratory evidence suggests that acupuncture induces multilevel regulation through complex mechanisms against cerebral ischemia, including: (1) neuroprotection, (2) collateral circulation reconstruction, (3) neuro-regeneration, and (4) modulating brain glucose metabolism and brain plasticity [73]. The data of acupuncture clinical trials for stroke treatment are summarized in Additional file 1: Supplimentary Table 2.

### Acupuncture methods in stroke

**Fig. 2:**
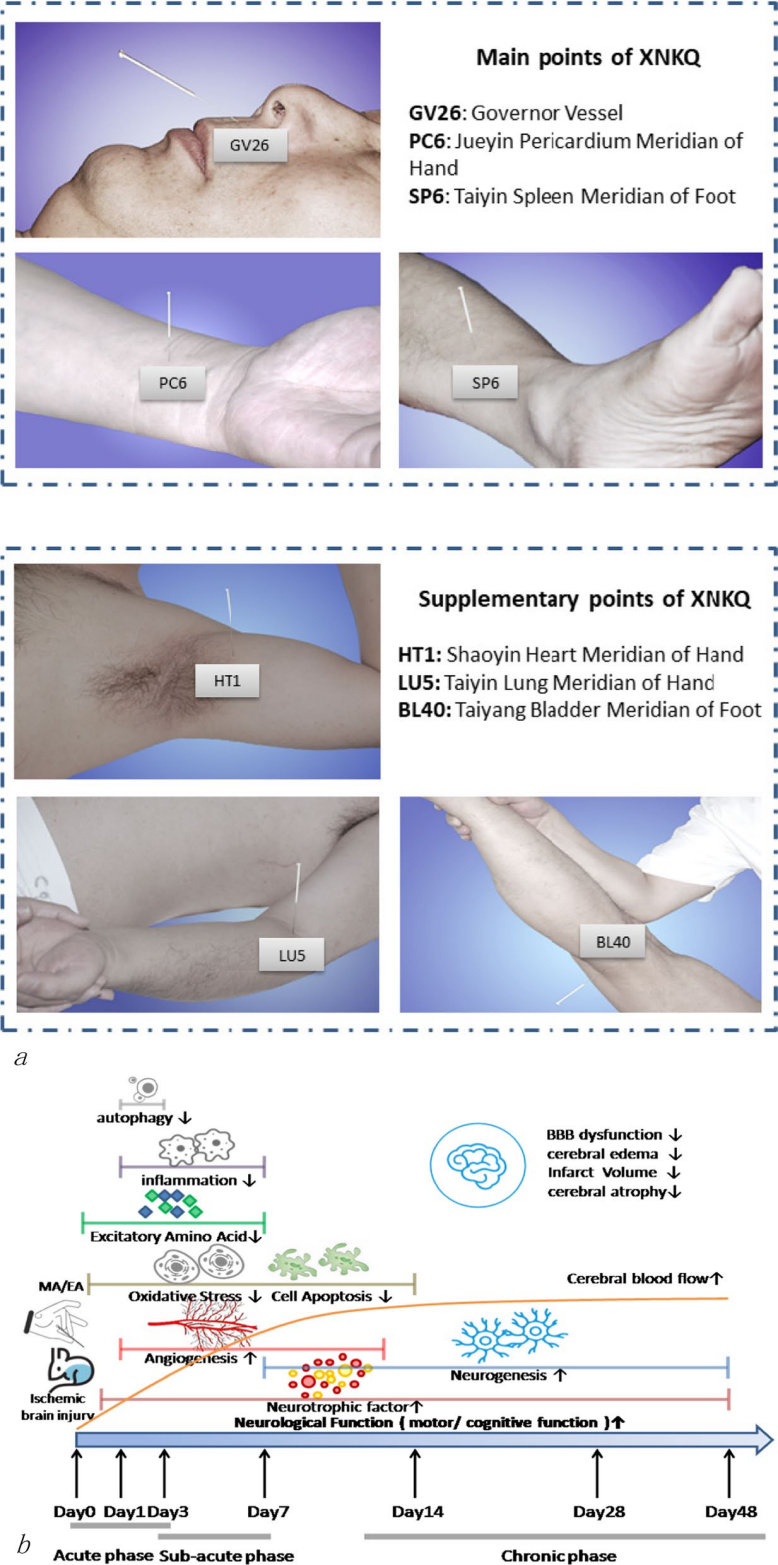
Acupuncture method and intervention on stroke. **a** XingNaoKaiQiao (XNKQ) protocol, an acupuncture method for ischemic stroke treatment, includeing stimulating the main points to induce resuscitation and to tonify the liver and kidney and the supplementary points to dredge the meridians. **b** Acupuncture intervention may be applied in any stage of stroke with various therapeutic effects that promote the development of collateral circulation and prevent further brain damage at the acute phase and stimulate endogenous neurogenesis at the post-acute phase. *BBB* blood–brain barrier, *MA/EA* manual acupuncture/electrical acupuncture

In acupuncture, specific points on the body meridians are called acupoints. When acupoints are stimulated by needles, vital energy inside meridians is excited and produces the effects of dredging meridians, resulting in harmonizing the internal environment, attenuating the pathogenic factors, and may finally relieving symptoms or curing diseases [104]. There are many methods of acupuncture to treat stroke, but broadly, it falls into two major categories based on the intervention styles and techniques: manual acupuncture (MA) or electroacupuncture (EA). Acupuncture interventions have been applied at any stage in stroke progression and recovery after the patient's vital signs are stabilized. The therapeutic acupoint selection, schedule and course duration are variable depending on the seriousness of the illness and intervention time [74–81] (Fig. 2).

### Neuroprotective effects

Unlike neuroprotective agents, acupuncture is a multi-target neuroprotective solution, which enhances endogenous neuroprotection functions to diminish brain edema and the volume of cerebral infarction, thus promoting the recovery of neural functions via the following therapeutic mechanisms.

#### Attenuating inflammation

Acupuncture can modulate inflammatory factors in brain tissues or peripheral blood that indirectly inhibit the activation of nuclear factor κB (NF-κB), reduce the synthesis and secretion of pro-inflammatory cytokines, such as TNF-α, Interleukin-6(IL-6), IL-8, IL-1β, and TNF-α, and at the meanwhile promote the secretion of anti-inflammatory cytokines IL-10. Acupuncture also attenuates the excessive activation of microglia, astrocytes, and macrophages [82–84].

#### Attenuating the excitatory amino acids (EEAs)-induced toxicity

Previous studies have shown that EA could reduce the accumulation of glutamate and aspartic acids in the topical cerebral infarct area and alleviate glutamate toxicity to neurons via regulating the expression of NMDARs and reducing Ca^2+^ influx in the hippocampus of cerebral ischemia/reperfusion injury rats [85, 86]. Furthermore, EA pretreatment could play a neuroprotective role by enhancing the expression of glutamate receptor subunit 2 (GluR2) in the hippocampus after global cerebral ischemia (GCI) reperfusion through cannabinoid CB1 receptors (CB1R) [87]. In addition, EA pretreatment could also increase the expression of glutamate transporter-1 (GLT-1) and inhibit the excessive release of glutamate in the striatum during ischemic-reperfusion brain injury [88]. GABA-mediated inhibition also plays a role in acupuncture-induced reduction of excitotoxicity. There is evidence that acupuncture stimulation at GV26 could decrease excessive release of glutamate induced by ischemia and maintain the endogenous inhibitory activity of GABA [89].

#### Inhibiting oxidative stress

Acupuncture therapy possesses the potential to reduce oxidative stress caused by cerebral ischemia, which may be related to the neuroprotective effect of acupuncture [90]. Existing research showed that acupuncture could block the production of pro-oxidative stress factors, such as nitric oxide (NO), inducible nitric oxide synthase (iNOS), malondialdehyde(MDA), superoxide anion, and oxidized glutathione in mitochondria, while promoting the production of antioxidant factors, including superoxide dismutase (SOD), CuZnSOD, MnSOD, cyclooxygenase (COX), and reducing glutathione [91, 92]. Additionally, acupuncture also inhibits synthesis of translocase of the outer mitochondrial membrane 40 and translocase of the inner mitochondrial membrane 17A, as well as the accumulation of amyloid β in brain mitochondria [91].

#### Diminishing cell apoptosis

Preclinical studies indicate that acupuncture could inhibit apoptosis, decrease infarct volume, and ameliorate neurological impairment via mechanisms mediated by different signaling pathways, such as PI3K/Akt and extracellular signal-regulated kinase (ERK)/c-Jun N-terminal kinase (JNK)/p38 [93, 94]. Acupuncture could also inhibit apoptosis after cerebral ischemia by increasing the expression of anti-apoptotic genes or protein B cell lymphoma 2 (Bcl-2), while reducing the expression of pro-apoptotic genes or proteins,  including BCL-2-associated X (Bax, capase 3, and caspase 9) [95, 96].

#### Regulating autophagy

EA pretreatment at GV20 decreases the expression of autophagy markers and the number of autophagosomes in the ischemic cortex [97]. EA at GV20, GV4, and ST36 decreases the level of mammalian target of rapamycin (mTOR) and increases the levels of autophagy-related protein Beclin1 and LC3, which can inhibit neuronal injury induced by autophagy during the reperfusion period of cerebral ischemia [98]. Furthermore, EA at LI11 and ST36 could protect against focal cerebral ischemia by inhibiting autophagosome formation and autophagy, which is mediated via the mammalian target of rapamycin complex 1-Unc-51-like kinase (mTORC1-ULK) complex-Beclin1 pathway [99].

### Vascular remodeling and angiogenesis

#### Modulating integrity of the BBB

Existing evidence indicates acupuncture might alleviate BBB dysfunction during ischemic stroke. During the acute stage of ischemic stroke, EA or MA could reduce BBB permeability and brain edema by increasing the expression of tight junction proteins ZO-1 and claudin-5 in the ischemic cortex, decreasing the expression of ROS generation, NADPH oxidase 4 (NOX4) and astrocytic aquaporin 4 (AQP4) in the peri-infarct area [100], and inhibiting expression of MMP-2 and MMP-9, AQP4 and APQ9, which are implicated in BBB permeabilization and destruction [101, 102].

#### Adjusting the CBF

Ischemic stroke results from the occlusion of a cerebral artery followed by severe disturbances in blood supply through micro-vessels to brain tissues. Acupuncture has been shown to increase CBF and improve microcirculation, which could potentially explain the beneficial effects of acupuncture on treating cerebral ischemia. EA may reduce vasoconstriction and improve blood supply in ischemic region by suppressing the expression of Angiotensin II and its receptor-mediated signaling pathway [103], and induce changes in cell proliferation-associated miRNA expression after stroke [104].

#### Promoting post-stroke angiogenesis.

EA increase the endothelial cell proliferation from as early as 12 h post-MCAO [105]. Meanwhile, EA intervention can alleviate the injury of microvascular ultrastructure of focal ischemic cerebral tissues and upregulate cerebral VEGF mRNA expression, suggesting a role of EA in protecting ischemic brain tissues by facilitating the angiogenesis of capillary vessels and thereby restoring the function of the damaged microvasculature [106]. EA also accelerates and promotes production of stromal cell-derived factor-1 (SDF-1) which further induces the mobilization of EPCs [107], and increases the level of angiogenesis promoting factors in MCAO rats, including bFGF, angiogenin (Ang)-1/2, PDGF-b, which promote vascular endothelial cell proliferation and the recovery of neurological function [108]. Furthermore, EA at GV26 promotes regional CBF on the infarcted and non-infarcted hemisphere and increases the number of blood vessels in areas of infarctions by upregulating von Willebrand factor and vascular endothelial cell proliferation [109].

### Neurogenesis

Acupuncture is potentially beneficial for post-stroke rehabilitation and is considered a promising preventive strategy for stroke. EA pretreatment or treatment after ischemic stroke generates neuroprotective and neuro-regenerative effects [110]. A systematic review showed that acupuncture enhanced endogenous neurogenesis including proliferation, migration, and differentiation of NSCs in experimental ischemic stroke models [111]. Different stimulation methods of acupuncture as well as the selection of acupoints lead to neurogenesis in different regions of the brain. EA treatment applied at GV20 and GV14 after MCAO may promote functional recovery by enhancement of proliferation and differentiation of NSCs in the hippocampus and SVZ of the ipsilateral hemisphere via the BDNF and VEGF signaling pathway [112]. Acupuncture stimulation on GV26 enhanced the “self-repairing” capacity of MCAO rats and alleviated neural functional damage by increasing the brain blood flow and the population of BrdU+, Nestin+, BrdU/nestin co-labeled immunofluorescence positive cells in penumbra and promoting the expression of nestin mRNA in cortex and hippocampus, which facilitates endogenous neurogenesis and may be associated with regulating GSK-3β and PP2A expression [113].

### Influencing factors and limitations of acupuncture

The National Institute of Health published a consensus statement that acupuncture may be useful as an adjunct treatment or an acceptable alternative to be included in a comprehensive management program for stroke care [114]. In recent years, clinical trials of acupuncture registered and carried out worldwide involve the evaluation of limb motor function, limb spasm and pain, swallowing function, activities of daily living, quality of life, cognitive function, depression, and anxiety state, etc., both in acute and recovery phase of ischemic stroke. Among them, two multicenter clinical trials showed that acupuncture was safe for the acute and subacute phase of ischemic stroke, reducing long-term mortality or disability rate and improving the neurologic deficits of patients [115, 116].

Shown from the available evidence, acupuncture plays varying roles in stroke treatment depending on the degree of cerebral ischemia. The efficacy is depending on the selection of acupoints and formula as well as the operator's manipulation skills, the timing of intervention, and the frequency of acupuncture sessions [117]. A multicenter prospective cohort study showed that early intervention produced better effects on the disability and motor dysfunction of patients with cerebral infarction and limb dysfunction [117]. However, more high-quality clinical evidence is still needed to build the consensus on the frequency and interval of acupuncture intervention in different phases of ischemic stroke.

Diversified acupoint selection, prescription, and stimulation parameters in the acupuncture treatment plan are some of the major barriers between laboratory research and clinical applications of acupuncture. The therapeutic mechanism of acupuncture on focal cerebral ischemia involves multiple targets and signaling pathways to exert acupuncture’s neural protective effects, and this complexity increases the difficulty of setting up negative and placebo control groups. Lack of positive control and direct efficacy indices (such as infarct volume) also influence the evaluation of acupuncture efficacy. To obtain more reliable clinical effects, quantitative and standardized managing acupuncture treatment is necessary to be established based on the clarification of the ‘acupuncture method—neural electrical signal code—acupuncture effect’, and the ‘dose-effect’ relationship.

Although the simple operation and no facility-dependent properties are the advantages of acupuncture, acupuncture may has limited potential for sustainable technical improvement as a thousand-year-old well-developed clinical treatment method. Only a theoretical breakthrough based on modern biomedical analysis can optimize the current therapy protocols or the developement of a joint therapeutic methodology could significantly benefit the patients.

## Combination of stem cell therapy and acupuncture

**Fig. 3:**
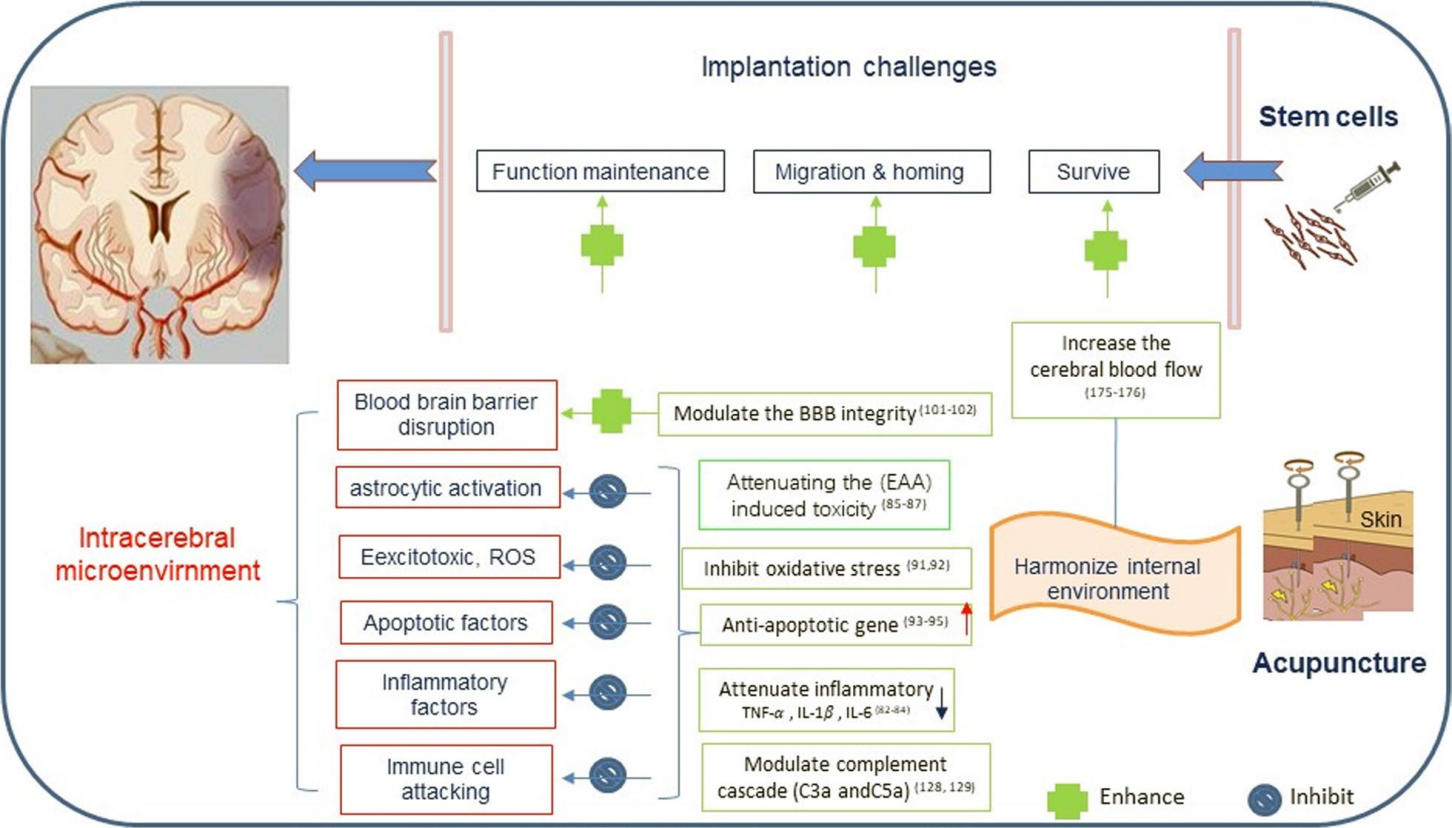
Acupuncture may enhance the efficacy of cell implantation. Acupuncture may increase the survival rate, migration and homing ability of the implated cells and maintain cell functions by increasing the cerebral blood flow, modulating the blood brain barrier (BBB) integrity, and attenuating the excitotoxicity and inflammatory responses. *EAA* excitatory amino acid, *IL* interleukin, *ROS* reactive oxygen species, *TNF* tumor necrosis factor

Stem cell transplantation and acupuncture are two individual types of multi-targeted therapies for ischemic stroke patients via different mechanisms and pathways. Acupuncture mainly modulates the microenvironment and activates endogenous restoration activities, while implantation of stem cells exerts its effect via exogenous stimulation of cell activities and/or direct integration for neuro-regeneration. Preclinical studies provided evidence that the combined stem cell transplantation and acupuncture treatment can significantly improve the neurological outcomes compared to a single type of therapy through three interactive mechanisms: (1) compensatory effects, where acupuncture increases the survival rate, migration, and homing ability of implanted cells; (2) enhancing effects that modulate the inflammatory response and oxidative stress; (3) synergistic effects that increase the regeneration ability of endogenous and exogenous NSCs [118, 119] (Fig. 3).

Compensatory effects—Stem cells facilitate the recovery and reconstruction of neurovascular unit and networks, as well as support the proliferation of endogenously activated NSC populations. Acupuncture can maintain normal stem cell functions and stimulate their participation in neural regeneration by increasing cerebral blood flow and regulating chemokines and various cell signal transduction pathways.

Local microenvironment and immune responses are the decisive factors affecting the in vivo fate and function of implanted stem cells. The chemokines and cytokines secreted locally in infarct site, such as SDF-1, SCF, and VEGF, can not only promote the mobilization and homing of implanted cells to the lesions because of stem cells' chemotaxis characteristics, but also influence the damaged sites by recruiting the exogenous stem/progenitor cells and affecting their functions [120]. Our previous studies have found that acupuncture rebuilds or repairs the signal network by regulating the expression of stem cell microenvironment-related cytokines, which aid in maintaining the interaction and signal connection between transplanted stem cells and neighboring cells, therefore, promoteing the survival and proliferation of homed stem cells in the impaired brain tissue [119].

Passive entrapment in the microvascular system is one of the major stem cell physical localization mechanisms [228, 229]. Migrating and homing ability of implanted stem cells have been linked with the improved local blood flow (physical and physiological influences) and the administration of relative homing factors (biochemical influences) by numerous studies [121–123]. Both animal and clinical studies have proved that acupuncture can increase the cerebral blood flow and cerebral microcirculation by regulating the expression of angiogenesis-related factors, which result in a more conducive microenvironment for the survival of transplanted stem cells and the regeneration of NVUs at the infarct site [103].

Enhancing effect**—**Stem cells produce neurotrophic and immunoregulatory factors via paracrine mechanisms, which could potentially enhance the neuroprotective effect of acupuncture. However, the low homing efficiency and survival rate of the systemic administrated stem cells limit their therapeutic efficacy in ischemic stroke treatment. Although the stereotaxic implanted NSCs have displayed a better survival rate, they showed very limited therapeutic effects because there is no stroma reserved in the liquefactive necrosis lesion site [124, 125]. Acupuncture can modulate the brain microenvironment and reduce the cell loss caused by excitotoxicity and oxidative stress, which could  further enhance cell therapy efficiency.

Both stem cell transplantation and acupuncture displayed immunoregulatory and antioxidative stress ability through different mechanisms. In an endometrial injury animal study, Xia, et al. reported that EA elevated the expression of endometrial surface chemokine, activated the SDF-1/CXCR4 axis, which resulted in enhanced migration and paracrine effects of BMSCs. Improved histological outcome, vibrant cell activity, and increased cytokine level were detected within the endometrial damaged area, resulting in a better embryo implantation rate [126].

The complement system is able to modulate the inflammation and immune responses, which hae been found indirectly influencing the cytotherapy outcomes [127]. The anaphylatoxins C3a and C5a, which are produced by complement components C3 and C5, are the human MSC chemo-attractants that direct the cell migrating and homing to the injured site when their C3a and C5a receptors (C3aR and C5aR) coupled to the MSC G_1_-protein. The resilience of the implanted MSCs to oxidative stress and ROS is enhanced by C3aR and C5aR binging, which results in an increased survival rate and normal cellular functions maintenance [128]. Chen, et al. reported that acupuncture downregulated the pro-inflammatory cytokine TNF-α and IL-1β, stimulated the release of complements, such as C3a and C5a, as well as the secretion of cytokines SDF-1 and TGFβ-1, which played a synergistic immunomodulatory effects when combined with stem cell-seeded cryogel/hydrogel biomaterials for treating the diabetic skin wounds [129].

Synergistic effect**—**Acupuncture can activate a limited number of endogenous NSCs while intracerebral transplanted NSCs are able to differentiate into several types of neural cells directly involved in functional recovery of the damaged brain tissues.

The combined intervention of EA and HUCB-MSC transplantation showed a synergetic effect on upregulating VEGF expression and inhibited the cellular apoptosis in the cerebral ischemic penumbra of the ischemic infarct core [130, 131]. Furthermore, Kim, et al. reported that elevated autologous NSC proliferation, BDNF, and neurotrophin-4 expression, and higher activation of the transcription factor cAMP response element-binding protein are detected in the brain of the combination therapy group in MCAO mice, indicating the combination therapy has more capability in neurotrophic factor modulation (Table 2). Combination therapy has been proved to be more advantageous than simple cell transplantation because it has a synergistic effect in co-regulating neurotrophic factors in the brain, promoting angiogenesis, inhibiting cell apoptosis, and promoting nerve function recovery of cerebral ischemia rats.

**Table 2:** The combination of cytotherapy and acupuncture in cerebral ischemia treatment

References	Cytotherapy and acupuncture intervention	Control group	Effect index	Comparison of effects between groups	Mechanism index
[130]	HUCB-MSCs (1 × 10^6^/10 µl, intracranial transplantation); EA (GV26, GV20, GV14, CV24, CV4, CV6; 30/100 Hz/5 V; 20 min; 7, 14, 28 days)	PBS group	Modified neurological severity score	EA + HUCB-MSCs > HUCB-MSCs > PBS	VEGF-positive cells↑
[131]	HUCB-MSCs (1 × 10^6^/10 µl, intracranial transplantation); EA (GV26, GV20, GV14, CV24, CV4, CV6; 30/100 Hz/5V; 20 min; 7, 14, 28 days)	PBS group	Pathological lesion	EA + HUCB-MSCs > HUCB-MSCs > PBS	Cellular apoptosis↓
[118]	mBMSC (1 × 10^5^/5 µl, intracranial transplantation); EA (GV14, GV20, 2Hz/2V; 20 min; 12 days)	MCAO group	Motor and cognitive dysfunctions Atrophic volume	mBMSC + EA > EA > mBMSC > MCAO	mBDNF↑, NT4↑ cAMP↑, pCREB↑ Proliferation of neural progenitor cells↑
[132]	TrkB-MSCs (1 × 10^6^/2 μl, intracranial transplantation); EA (GV14, GV20, 2 Hz/2 V; 20 min; 10–22 days)	PBS group, MSCs group, MSCs + EA group	Motor and cognitive function	TrkB-MSCs + EA > TrkB-MSCs > MSCs + EA > MSCs > PBS	BDNF↑, NT4↑ Survival, differentiation and migration of TrkB-MSCs into mature neuronal cells↑ Activation of BDNF/NT4/TrkB Signaling pathway

A later study from the same research group found that stereotactic injection of BDNF/NT4 receptor tropomyosin receptor kinase B (TrkB) gene-transfected BM-MSCs (TrkB-MSCs) combined with EA treatment in MCAO mice showed better motor function improvement than the single therapy. The histological analysis revealed that the combined therapy group resulted in more TrkB-MSCs differentiating into neural cells, which suggests EA displayed an enhancing role in the composition therapy by increasing the expression of neurotrophic factors, BDNF and NT4, to activate the residential NSCs as well as to promote the differentiation of TrkB-MSCs into matured neurons [118, 132].

## Conclusion and prospects

**Fig. 4:**
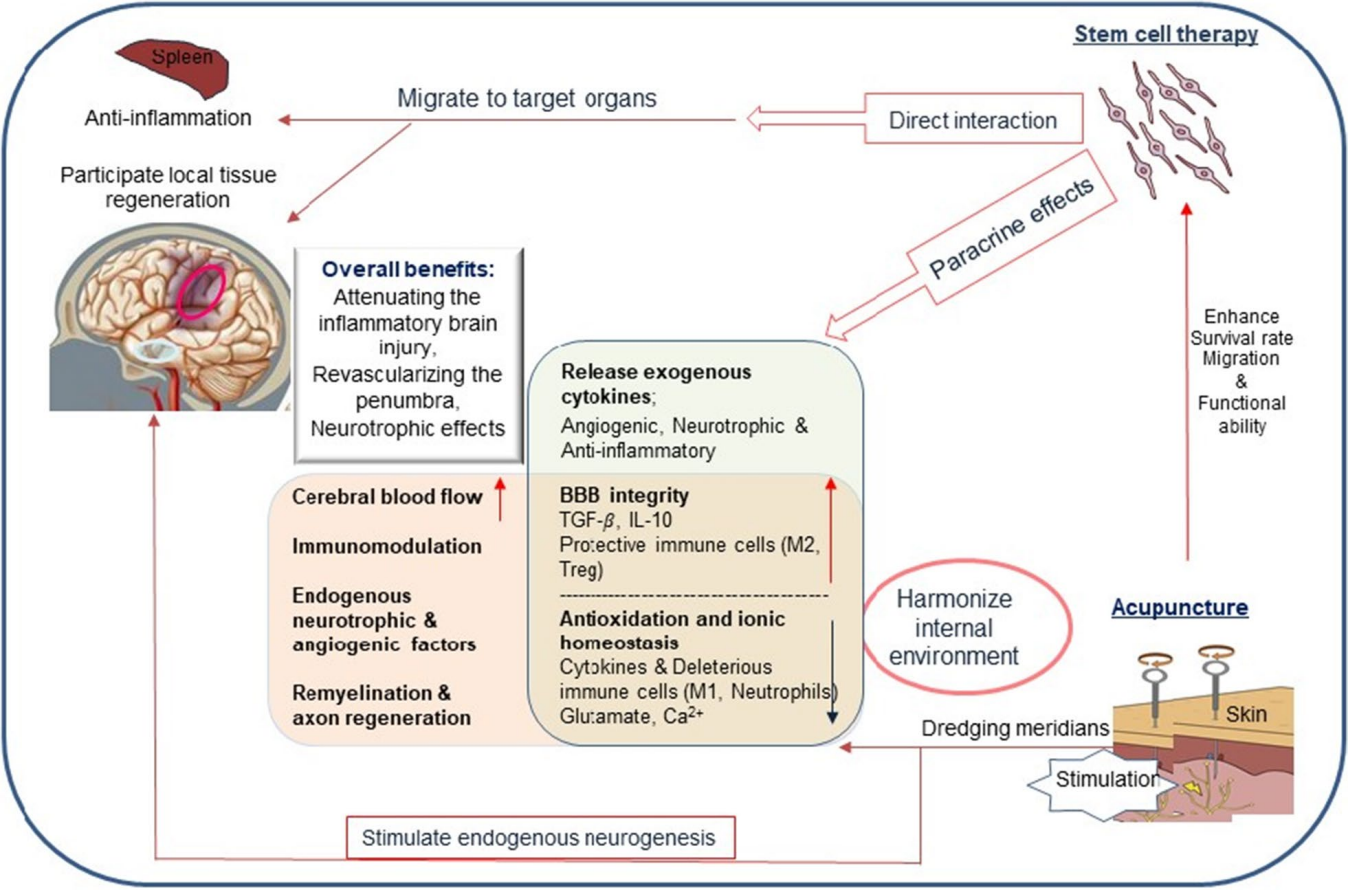
The potential benefits of a stem cell therapy and acupuncture combination treatment for ischemic stroke. The implanted cells may protect the brain mostly depending on their paracrine effects and some peripheral immunomodulation activity. The acupuncture will mainly harmonize the internal environment, stimulate endogenous neurogenesis, and help the implanted cells to overcome some in vivo challenges. *IL* interleukin, *TGF-β* transforming growth factor beta

Preclinical and clinical study revealed that both stem cell transplantation and acupuncture can improve the recovery and rehabilitation of ischemic stroke to a certain extent and acquire multi-target advantages compared to the currently available clinical treatments. A combined therapy may increase the implanted stem cells' survival, homing, and functional differentiation rate, and benefit ischemic stroke patients by enhancing and synergizing the effects of individual treatments and compensating for the deficits of each therapy compared to when each therapy is administered on its own (Fig. 4).

### Prospects

Stem cell therapy offers more dynamic interventions compared to drugs. The convenient, low-cost, and safe acupuncture treatment is an ideal supplementary therapy for stem cell therapy in ischemic stroke management. On the premise of quantified acupuncture methodology, the benefits of combined therapy should be evaluated by randomized, double-blind clinical trials that group ischemic stroke patients by age, obstruction location, primary cause, and stage to narrow down and examine specific indications. Novel molecular biological detections, cell labeling, and imaging techniques should be able to disclose more neuroprotection, neuro-regeneration, and anti-inflammatory solution as well as other mechanisms of stroke therapies.

## Supplementary Information

**Additional file 1.** Supplementary Table 1. Supplementary Table 2.

## Data Availability

Not applicable.

## Abbreviations

Ang: Angiogenin
AIF: Apoptosis-inducing factor
FGF-2: Basic fibroblast growth factor
Bcl-2: B cell lymphoma 2
BBB: Blood–brain barrier
BMP: Bone morphogenetic protein
BDNF: Brain-derived neurotrophic factor
ERK/JNK/p38: C-Jun N-terminal kinase (JNK)/p38
CNS: Central nervous system
CBF: Cerebral Blood Flow
RCTs: Controlled trials
EA: Electrical acupuncture
EAA: Excitatory amino acid
ESCs: Embryonic stem cells
ENOS: Endothelial nitric oxide synthase
EPCs: Endothelial progenitor cells
EGF: Epidermal growth factor
EPO: Erythropoietin
ERK: Extracellular signal-regulated kinase
GDNF: Glial‐derived neurotrophic factor
GFAP: Glial fibrillary acidic protein
HSCs: Hematological stem cells
HSPGs: Heparan sulfate proteoglycans
4-OHT: 4-Hydroxytamoxifen
iPSCs: Induced pluripotent stem cells
IκB: Inhibitor of NF-κB
IL: Interleukin
IA: Intraarterial
IT: Intrathecal
IP: Intraperitoneal
IV: Intravenous
Jag1: Jagged-1
LPS: Lipopolysaccharide
Mtor: Mammalian target of rapamycin
MA: Manual acupuncture
mmps: Matrix metalloproteinases
mscs: Mesenchymal stem cells or multipotent stem cells
MCAO: Middle cerebral artery occlusion
mncs: Mononuclear cells
NK: Natural killer cells
NGF: Nerve growth factor
npcs: Neural progenitor/precursor cells
NRG: Neural regeneration
nscs: Neural stem cells
neun: Neuronal nuclei
NO: Nitric oxide
NMDA: *N*-Methyl-d-aspartate
(ngr)/rhoa: Nogo-A/Nogo receptor
Dll4: Notch-ligands Delta-4
Olig-2: Oligodendrocyte
OGD: Oxygen–glucose deprivation
PDGF: Platelet-derived growth factor
PE: Paracrine effect
pbmcs: Peripheral blood mononuclear cells
PI3K/Akt: Phosphatidylinositol 3-kinase/protein kinase B
ROS: Reactive oxygen species
rcts: Randomized controlled trials
Tregs: Regulatory T cells
ROCK: Rho-associated protein kinase
SVZ: Subventricular zone
SGZ: Sub-granular zone
SHH: Sonic hedgehog
SDF-1α: Stromal cell-derived factor-1α
TCM: Traditional Chinese Medicine
TGF-β: Transforming growth factor beta
TREM2: Triggering receptor expressed on myeloid cells 2
TNF: Tumor necrosis factor
UCB: Umbilical cord blood
VEGF: Vascular endothelial growth factor
XNKQ: XingNaoKaiQiao
