# Supplementary material for: Combination of stem cell therapy and acupuncture to treat ischemic stroke: a prospective review
Source: Stem Cell Res Ther. 2022 Mar 3;13:87. doi: 10.1186/s13287-022-02761-y (PMC8896103; doi:10.1186/s13287-022-02761-y)
Supplement: Supplementary file 1 — Additional file 1. Supplementary Table 1. Supplementary Table 2. [file 13287_2022_2761_MOESM1_ESM.docx]

**Supplementary Tables:**

Supplementary Table 1. Clinical trials of cell therapy for stroke

| **Cell Type** | **Study Title** | **Conditions** | **Interventions** | **Locations** | **Status** |
| --- | --- | --- | --- | --- | --- |
| MSC | Intravenous Autologous Mesenchymal Stem Cells Transplantation to Treat Middle Cerebral Artery Infarct | Middle Cerebral Artery Infarction | Standard medical care  Biological; Autologous bone marrow-derived MSCs | Malaysia | Unknown  (Phase II; 50)  2012 – 2016 |
| MSC | Human Umbilical Cord Mesenchymal Stem Cell Therapy for Cerebral Infarction Patients in Convalescent Period. | Cerebral Infarction | Allogeneic umbilical cord MSCs; Aspirin Enteric-coated Tablets & Atorvastatin Calcium | China | Not yet recruiting |
| MSC | The Safety and Efficacy of Human Umbilical Cord Mesenchymal Stem Cells in the Treatment of Acute Cerebral Infarction | Cerebral Infarction | Allogeneic umbilical cord MSCs (SCLnow 19# - Sclnow Biotech); Aspirin Tablet | China | Not yet recruiting  (Phase I; 40) |
| MSC | Study of Human Placenta-derived Cells, PDA001- (cenplacel-L), to Evaluate the Safety and Effectiveness for Patients With Ischemic Stroke | Stroke, Acute Middle Cerebral Artery Stroke, Posterior Cerebral Artery Stroke | Human Placenta-Derived Cells, PDA001- (cenplacel-L) –  Cytomedix, Inc. | United States | Terminated  (Phase II; 44) |
| MSC | Study of ALD-401 Via Intracarotid Infusion in Ischemic Stroke Subjects | Stroke, Ischemic Stroke, Stroke in Middle Cerebral Artery | ALD-401 (a unique population of bone marrow-derived adult stem cells) | United States | Unknown  (Phase II; 100) |
| MSC | Evaluation of the Safety and Potential Therapeutic Effects After Intravenous Transplantation of Cordstem-ST in Patients With Cerebral Infarction | Cerebral Infarction | Cordstem-ST (Human umbilical cord derived MSC therapy - CHA Biotech) | Korea | Active, not recruiting  (Phase I / IIa; 18) |
| MSC | Cord Blood Infusion for Ischemic Stroke | Stroke | Allogeneic umbilical cord blood | United States | Completed  (Phase I; 10)  2015 – 2017 |
| MSC | Autologous Bone Marrow Stromal Cell and Endothelial Progenitor Cell Transplantation in Ischemic Stroke | Stroke Infarction, Middle Cerebral Artery | Autologous BMSCs vs. Autologous EPCs | China | Unknown |
| MSC | A Study of Modified Stem Cells in Stable Ischemic Stroke | Chronic Ischemic Stroke | SB623 | United States | Completed  (Phase I / IIa; 18)  2011 – 2015 |
| MSC | A Study of Allogeneic Mesenchymal Bone Marrow Cells in Subjects With Ischemic Stroke | Ischemic Stroke | Allogeneic adult bone marrow MSCs | United States | Active, not recruiting |
| MSC | Study of Autologous Stem Cell Transplantation for Patients With Ischemic Stroke | Infarction, Middle Cerebral Artery | Autologous bone marrow transplantation | Brazil | Completed  (Phase I; 12)  2007 – 2011 |
| MSC | Study to Examine the Effects of MultiStem in Ischemic Stroke | Ischemic Stroke | MultiStem (human SCs from adult bone marrow or other nonembryonic tissue sources) - Athersys | United States | Completed  (Phase II; 134)  2011 – 2016 |
| MSC | Study of Allogeneic Umbilical Cord Blood Infusion for Adults With Ischemic Stroke | Stroke; Acute Stroke, Acute Brain Injury | Umbilical Cord Blood | United States | Recruiting  (Phase II; 100) |
| MSC | Ex Vivo Cultured Adult Allogenic MSCs in Ischemic Cerebral Stroke | Stroke | Ex vivo cultured adult allogenic MSCs | Malaysia | Withdrawn  (Phase I / IIa) |
| MSC | Regenerative Stem Cell Therapy for Stroke in Europe | Stroke | Adipose-Derived MSCs | France | Withdrawn |
| MSC | Perinatal Arterial Stroke Treated With Stromal Cells Intranasally | Perinatal Arterial Ischemic Stroke  Neonatal Stroke | MSCs | Netherlands | Not yet recruiting  (Phase I / IIa; 10) |
| MSC | Study of Modified Stem Cells (SB623) in Patients With Chronic Motor Deficit From Ischemic Stroke | Chronic Ischemic Stroke | SB623 Implant (2.5M; 5.0M) | United States | Active, not recruiting  (Phase II; 156)  2016 – 2019 |
| MSC | Study to Assess the Safety and Effects of Autologous Adipose-Derived Stromal Cells in Patients After Stroke | Stroke | MSCs (through internal carotid artery and intravenously) | Mexico | Withdrawn |
| MSC | A Clinical Trial to Study the Safety and Efficacy of Bone Marrow Derived Autologous Cell for the Treatment of Stroke | Stroke | Intrathecal transplantation of Autologous Stem Cells | India | Unknown  (Phase I / IIa; 50) |
| MSC | Stem Cell Therapy For Acute Ischemic Stroke Patients | Middle Cerebral Artery Infarction; Anterior Cerebral Artery Infarction | Bone Marrow MSCs | India | Completed  (Phase II; 120)  2009 – 2011 |
| MSC | Umbilical Cord Derived Mesenchymal Stem Cells Treatment in Ischemic Stroke | Stroke | Human umbilical cord MSCs | China | Unknown |
| MSC | Reparative Therapy in Acute Ischemic Stroke With Allogenic Mesenchymal Stem Cells From Adipose Tissue, Safety Assessment, a Randomised, Double Blind Placebo Controlled Single Center Pilot Clinical Trial | Ischemic Stroke | Allogenic MSCs from adipose tissue | Spain | Completed  (Phase II; 19)  2014 – 2017 |
| MSC | The STem Cell Application Researches and Trials In NeuroloGy-2 (STARTING-2) Study | Stroke, Ischemic | MSCs | Korea | Recruiting  (Phase III; 60) |
| MSC | Efficacy Study of CD34 Stem Cell in Chronic Stroke Patients | Stroke; Middle Cerebral Artery Infarction | Intercerebral implantation of Autulogous Stem Cells | Taiwan | Completed  (Phase II; 30) |
| MSC | Intravenous Stem Cells After Ischemic Stroke | Ischemia; Stroke | Autologous MSCs | France | Completed  (Phase II; 2) |
| MSC | Autologous Bone Marrow Mesenchymal Stem Cell Transplantation for Chronic Stroke | Stroke | Intravenous/intracerebral SC transplantation | China | Unknown |
| NSC | Implantation of Olfactory Ensheathing Cells | Infarction, Middle Cerebral Artery Ischemic Stroke, Stroke With Hemiparesis, Thromboembolic Stroke | Olfactory ensheathing cells | Taiwan | Unknown  (Phase I; 6)  2011 – 2013 |
| NSC | REGENESIS (US): A Phase IIb Prospective, Randomized, Double-blind, Placebo Controlled Study of NTx™-265: Human Chorionic Gonadotropin (hCG) and Epoetin Alfa (EPO) in Acute Ischemic Stroke Patients | Stroke | NTx™-265: rhCG, then rEPO | United States; Canada | Withdrawnl; Terminated  (Phase II; 30; 134) |
| NSC | Pilot Investigation of Stem Cells in Stroke Phase II Efficacy | Ischaemic Stroke; Cerebral Infarction; Hemiparesis; Arm Paralysis | CTX DP (20 million cells; intra-striatal injection) | United Kingdom | Completed  (Phase II; 23)  2014 – 2017 |
| NSC | Pilot Investigation of Stem Cells in Stroke | Stroke | CTX0E03 NSCs | United Kingdom | Active, not recruiting |
| NSC | Intracerebral Transplantation of Neural Stem Cells for the Treatment of Ischemic Stroke | Ischemic Motor Stroke, Chronic | NSI-566 | China | Active, not recruiting  (Phase I; 18) |

Supplementary Table 2 Clinical trials of acupuncture for stroke

| Study Title | Conditions | Interventions | Locations | Status |
| --- | --- | --- | --- | --- |
| Acupuncture Intervention to Improve Neurological Function and Anti-inflammatory Effect in Acute Ischemic Stroke(ANAIS) | Acute Ischemic Stroke  Acupuncture | Procedure: Traditional Acupuncture  Procedure: Minimal Acupuncture | China | Completed |
| Acupuncture Treatment on Motor Dysfunction in Stroke Patients | Stroke  Motor Disorders | Other: Acupuncture Treatment  Other: Rehabilitation Treatment | China | Active, not recruiting |
| Clinical Evaluation of Acupuncture on the Complications of Cerebral Vascular Accident | Stroke | Procedure: acupuncture(44mm in length and 32-gauge) | China | Terminated |
| Investigation of the Use of Acupuncture for the Treatment of Spasticity in Chronic Stroke Participants | Stroke | Other: Acupuncture  Other: Sham needle | Canada | Unknown † |
| A Clinical Trail of Acupuncture and Liu-Zi-Jue Exercise for Dysphagia in Post-stroke | Stroke | Combination Product: Chinese traditional rehabilitation | China | Recruiting |
| The Efficacy of Acupuncture on Patients With First Acute Ischemic Stroke, With Signs of Hemiplegia and Hemiparesis | Stroke | Device: real Acupuncture Needles  Device: sham Acupuncture Needles | Israel | Completed |
| Evaluating the Therapeutic Effect of Scalp Acupuncture Treatment for Motor Dysfunction in Ischemic Stroke Patients | Ischemic Stroke  Hemiplegia | Device: Scalp Acupuncture Treatment  Other: Rehabilitation treatment | China | Recruiting |
| Acupuncture and Computer-based Training to Improve Attention Deficits in Patients After Stroke | Stroke | Device: RehaCom  Device: Acupuncture | China  Germany  Switzerland | Unknown † |
| A Clinical Trail of Acupuncture and Herbs for Post-stroke Cogntive Impairment | Post-stroke Cognitive Impairment | Combination Product: Chinese traditional rehabilitation | China | Recruiting |
| The Establishment and Analysis of the Clinical Prediction Model of Acupuncture and Moxibustion for Stroke Recovery | Stroke | Procedure: Acupuncture | China | Unknown † |
| Scalp Acupuncture Combined rTMS on Brain White Matter Microstructure of Hemiplegic Patients With Stroke | Stroke | Other: the experimental group  Other: the control group |  | Completed |
| The Efficacy of an Acupuncture Protocol and Use of Taping in the Treatment of Spastic Upper Limb After Stroke | Stroke  Cerebrovascular Stroke | Procedure: Acupuncture  Other: Functional Taping  Other: Acupuncture and Functional Taping | Brazil | Completed |
| Acupuncture for Ischemic Post-stroke Depression | Post-stroke Depression | Device: acupuncture  Drug: placebo  Device: sham-acupoint acupuncture  Drug: Fluoxetine | China | Completed |
| The Effectiveness of Acupuncture Treatment for Post-Stroke Depression and Anxiety Disorder. | Post-stroke Depression  Anxiety Disorders | Procedure: Acupuncture Treatment  Other: Conventional Rehabilitation Programme | Turkey | Completed |
| Scalp Acupuncture for Dyskinesia After Ischemic Stroke | Ischemic Stroke | Other: Scalp acupuncture  Other: Conventional rehabilitation | China | Active, not recruiting |
| Can Acupuncture Treat Post-stroke Depression? | Stroke  Depression  Stroke Sequelae | Procedure: Dense cranial electroacupuncture stimulation  Procedure: Body acupuncture  Drug: Antidepressant  (and 2 more...) | China | Completed |
| Acupuncture in Stroke Rehabilitation | Stroke | Other: Acupuncture and Strengthening  Other: Strengthening Alone | United States | Terminated |
| Lu's Acupuncture and Moxibustion Treatment on Stroke | Ischemic Stroke  Paralysis | Procedure: Acupuncture  Procedure: Rehabilitation | China | Recruiting |
| Acupuncture for Stroke Recovery | Systematic Acupuncture | Procedure: Acupuncture Treatments | United States | Terminated |
| Using fMRI and sEMG to Evaluate the Effects and Mechanism on Abdominal Acupuncture Combined With Upper Limb Rehabilitation Training on Brain Plasticity of Hemiplegic Patients With Stroke | Brain Ischemia  Hemiplegic Patients WithStroke  Image Science  (and 2 more...) | Other: Abdominal acupuncture  Other: Sham abdominal acupuncture | China | Recruiting |
| Evaluating the Therapeutic Effect of Acupuncture on Acute Ischemic Stroke Patients | Acute Stroke | Other: acupuncture  Device: acupuncture  Device: Acupuncture |  | Completed |
| Acupuncture on Post-Stroke Overactive Bladder | Overactive Bladder | Other: Traditional Acupuncture  Other: Usual Care | China | Completed |
| Acupuncture for Post-stroke Shoulder-hand Syndrome | Shoulder-hand Syndrome | Device: rehabilitation  Device: Acupuncture | China | Unknown † |
| Acupuncture Lowering Blood Pressure for Secondary Prevention of Stroke | Cerebral Infarction | Device: acupuncture | China | Unknown † |
| Acupuncture for Hemiplegic Shoulder Pain | Shoulder Pain in Hemiplegic Side AfterStroke | Procedure: acupuncture  Procedure: TENS  Procedure: sham acupuncture | China | Completed |
| Acute Ischemic Apoplexy Syndrome Specificity and Acupuncture Intervention Research | Acute Ischemia | Other: Acupuncture Treatment  Other: Regular Treatment |  | Not yet recruiting |

† Study has passed its completion date and status has not been verified in more than two years.
